# A Simple Method for Fabrication of Microstructures Using a PDMS Stamp

**DOI:** 10.3390/mi7100173

**Published:** 2016-10-01

**Authors:** Hun Lee, Domin Koh, Linfeng Xu, Sindhu Row, Stelios T. Andreadis, Kwang W. Oh

**Affiliations:** 1 Sensors and MicroActuators Learning Laboratory (SMALL), Department of Electrical Engineering, University at Buffalo, State University of New York, Buffalo, NY 14260, USA; hlee24@buffalo.edu (H.L.); dominkoh@buffalo.edu (D.K.); linfengx@buffalo.edu (L.X.); 2 Department of Chemical and Biological Engineering, University at Buffalo, State University of New York, Buffalo, NY 14260, USA; sindhuro@buffalo.edu (S.R.); sandread@buffalo.edu (S.T.A.)

**Keywords:** PDMS microwell, PDMS microwell transfer, PDMS stamp, cell-to-cell study

## Abstract

We report a simple method to fabricate PDMS (polydimethylsiloxane) microwell arrays on glass by using a PDMS stamp to study cell-to-cell adhesion. In the cell-to-cell study, a glass substrate is required since glass has better cell attachment. The microwell arrays are replicated from an SU-8 master mold, and then are transferred to a glass substrate by lifting the PDMS stamp, followed by oxygen plasma bonding of the PDMS stamp on the glass substrate. For the cell-to-cell adhesion, four different types of PDMS arrays (e.g., rectangle, bowtie, wide-rhombus, and rhombus) were designed to vary the cell-to-cell contact length. The transfer success rates of the microwell arrays were measured as a function of both the contact area of the PDMS and the glass substrate and the different ratios between the base polymers and the curing agent. This method of generating the microwell arrays will enable a simple and robust construction of PDMS-based devices for various biological applications.

## 1. Introduction

The microwell array has become an essential analysis tool for various biological and chemical applications [1]. It is important to develop efficient fabrication methods for the microwell arrays, which can offer lower costs and simpler production methods [1,2,3]. Particularly, PDMS (polydimethylsiloxane) microwell arrays have been employed in manipulating and culturing cells for studying the individual behavior of cells in a microenvironment. Due to the fact that PDMS can be easily fabricated by using soft-lithography techniques and because it possesses many advantageous properties such as optical transparency, a non-toxic nature, and biocompatibility, it is considered to be a good material for various biological experiments [4,5]. In the early stages of cell-to-cell interaction studies, Petri dishes were used to form cell-to-cell contact [5]. In cell-to-cell studies, cells must be attached on the substrate, and glass is a good substrate for attaching cells because cells like hard surfaces [6]. 

However, the ability to observe the cells that grow in the dish is limited due to the difficulty in growing and pairing cells for cell-to-cell study. Under conventional cell culture conditions, it is difficult to manipulate the cell position, spreading, shape and density. For a controlled cell culture environment, a PDMS stamping (or imprinting) method has been widely used since it can easily print micropatterns on a substrate of interest [7,8,9]. This technology can offer a cell culture environment with well-controlled sizes, shapes, and positions on a substrate, thus providing a useful tool for cell studies. Gray et al. demonstrated a surface patterning method with cell-adhesive molecules using the PDMS stamping method for cell-to-cell contact studies [10]. Despite its huge potential, the contact printing method by the PDMS stamp should be carefully controlled to avoid deformation of patterns limiting the robust fabrication of the device for testing. With a similar approach, Nelson et al. utilized agarose micropatterns based on the PDMS stamp method on a glass substrate for studying cell-to-cell contact [11]. Its limitation is that, using this method, creating thick and high density patterns is challenging and, also, air bubbles can be easily trapped during the filling process of the agarose.

Here we demonstrate a simple fabrication method for creating the PDMS microwell arrays by transferring the patterns of the PDMS stamp onto a glass substrate. This method overcomes the limitations of the conventional PDMS imprinting method since it uses cured PDMS micropatterns only. Cured PDMS allows negligible volume shrink in microwell transfer because it is solid and the fact that no extra chemical treatment is necessary, just oxygen plasma treatment for the bonding during the transfer process, allows low-cost and quick fabrication of the devices. The handling of the device is easy and volume shrink is not as severe as when using agarose, without deformation of the pattern.

## 2. Methods/Experiment

### 2.1. Microarrays and Microfluidic Channel Design

**Figure 1:**
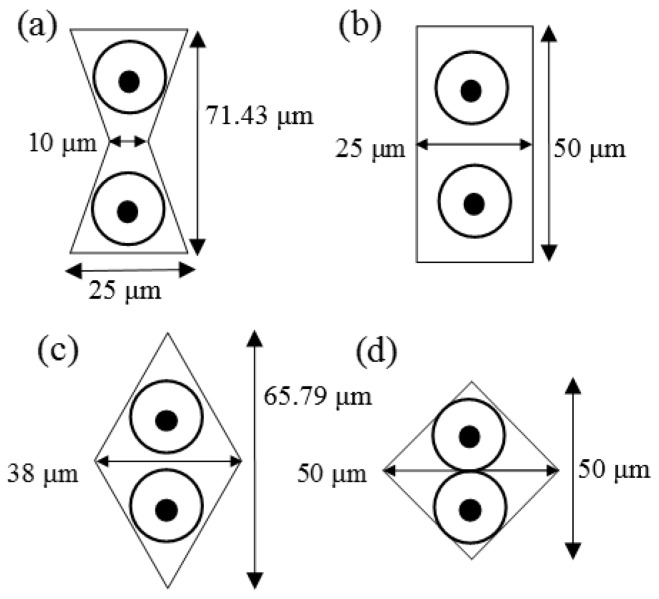
Schematic illustration of the four different types of microwell arrays to trap two cells for cell-to-cell contact. Each circle in each well represents the trapped cell in each well. The pattern size for all the shapes is fixed at 1250 μm^2^. (**a**) Bowtie; (**b**) Rectangle; (**c**) Rhombus; (**d**) Wide-rhombus shape.

For cell-to-cell interaction study using the suggested method, four different types of microwell arrays were designed to vary cell-to-cell contact length and to study the cell-to-cell interaction study. All types microwell have the same area, 1250 μm^2^, but different cell-to-cell contact length. The diameter of each microwell is in Figure 1. Pairs of cells will be trapped in each well like circles in each well in Figure 1. By changing the width of the centre of well, the contact area of each cell can be easily controlled.

**Figure 2:**
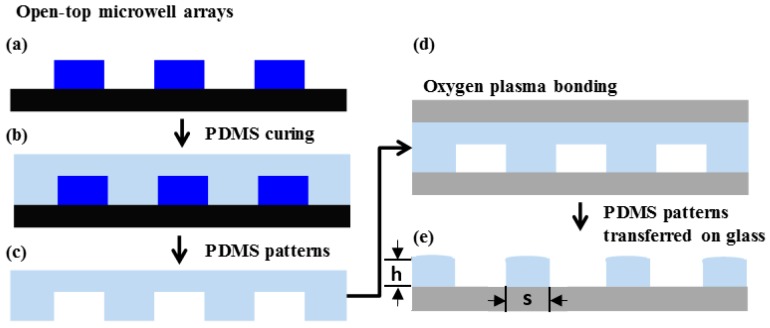
Schematic illustration of the fabrication process on the glass slide. (**a**) The microwell arrays are formed on a silicon wafer by using standard photolithography. (**b**) The PDMS (polydimethylsiloxane) is poured onto the mold and cured. (**c**) The replicated PDMS is peeled off from the mold. Then both sides of PDMS were treated with oxygen plasma to be bonded to glass substrates, as shown in (**d**). (**d**) Both sides of PDMS are bonded by two glass slides, then top glass substrate is removed with PDMS microwell array left on the bottom glass slide. (**e**) By applying the mechanical force to the top glass slide, the patterns can be easily transferred to the bottom glass.

The size of the PDMS microwell array stamp was 5 mm × 5 mm and the microwell arrays of each pattern has a 10 μm spacing (*s* in Figure 2) between patterns which is the width of the PDMS microwell. The heights of all patterns are 20 μm (*h* in Figure 2). The PDMS patterns with 20 µm thickness were designed and fabricated on the glass substrate for the cell-to-cell adhesion studies. By conventional soft-lithography, four different types of microwell arrays (e.g., rectangle, bowtie, wide-rhombus, and rhombus) were replicated from a master mould as shown in Figure 1. The density of patterns and contact surface area between the patterns and the glass are listed in Table 1.

### 2.2. Fabrication

The fabrication process of PDMS microwell arrays on a glass substrate is illustrated in Figure 2. The 20-µm-thick master mould (*h* in Figure 2) was initially fabricated through photolithography process using an SU-8 negative photoresist (SU-8 2015, Micro-Chem Corp., Newton, MA, USA) [12], and then it was treated with hexamethyldisilazane (Sigma Aldrich, Saint Louis, MO, USA) in a vacuum chamber for 1 h to easily peel off the PDMS stamp from the mold (Figure 2a). PDMS materials were made using pre-polymer and curing agent (Sylgard 184, Dow Corning Co., Midland, MI, USA), varying the mixing ratio from 5:1 to 20:1, from more stiff to less stiff. To mould the PDMS against the master, the mixture of pre-polymer and curing agent was carefully poured onto the SU-8 master mould and cured at 65 °C for 30 min (Figure 2b). The molded PDMS replica was peeled off and both sides of the PDMS was bonded irreversibly on two glass substrates by oxygen plasma treatment using plasma cleaner PDC-32G (Harrick Plasma, Ithaca, NY, USA) at power of 18 W (Figure 2c,d). Then the PDMS was lifted from the bottom glass substrate which leaving PDMS microwell array on the substrate (Figure 2e). The process of PDMS microwell array transfer on glass substrate take about 40 min excluding the fabrication process of the SU-8 master mold, which takes about 2 h including hexamethyldisilazane treatment.

### 2.3. Cell-to-Cell Study 

For the cell studies using the microwell arrays, human BM-MSCs (bone marrow mesenchymal stem cells) between passage 4 and 8 were trypsinized from normal culture conditions (Dulbecco’s modified Eagle medium containing 10% fetal bovine serum) then plated onto the microwell arrays on the glass substrate which were placed in six-well plates. For the experiments, cells were trypsinized and counted using a standard hemacytometer. The cells were then used in each pattern; cells settled by gravity into the microwell arrays, and were allowed to attach for 48 h. Following this, cells trapped in the microwell arrays were fixed using 4% paraformaldehyde, blocked with 5% goat serum for 1 h and exposed to primary antibody αSMA (α-smooth muscle actin, Sigma-aldrich, St. Louis, MI, USA) in 1:100 dilution. After incubation overnight at 4 °C, corresponding secondary antibodies were used in 1:200 dilution for 1 h at room temperature. Samples were counterstained with Hoechst 33342 dye (EMD Millipore Laboratory Chemicals, Billerica, MA, USA; 10 mg/mL; 1:200 dilution; 5 min at room temperature) for nuclei. Images were obtained using a Zeiss Axio-observer (Zeiss, Pleasanton, CA, USA) and analyzed by Image J software (NIH, Bethesda, MA, USA, https://imagej.nih.gov/ij/).

## 3. Result/Discussion

**Figure 3:**
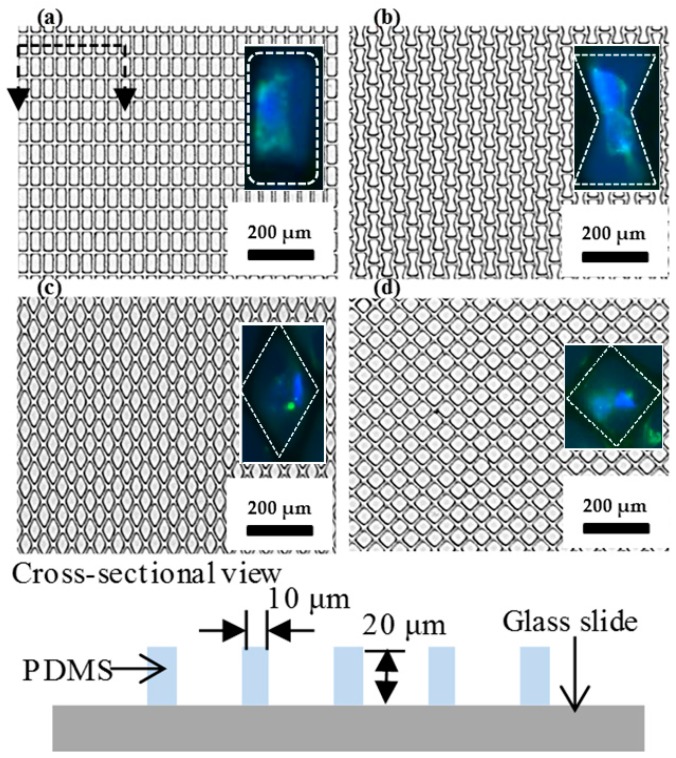
Images of the microwell arrays on the glass substrate. The microwell arrays have a 20-µm-thick and 10 µm gap among the patterns. Trapped cells in microwells were fluorescent-dyed and showed on the right side in (**a**,**b**). (**a**) Rectangle. Dashed line is illustrated on the bottom and it is a side-view of the PDMS pattern glass slide. The *h* of the PDMS is 20 µm and *s* is 10 µm; (**b**) Bowtie; (**c**) Rhombus; (**d**) Wide-rhombus shape.

**Figure 4:**
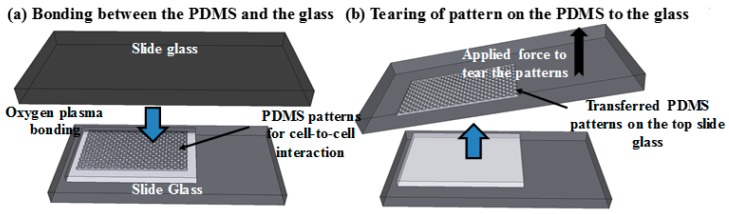
The illustration of peeling off PDMS after bonding. (**a**) Bonding two glass substrates to each side of the PDMS layer. (**b**) Peeling off to transfer patterns by applying force in the same direction. In this illustration, the patterns are transferred on the top slide but it is just for convenience (transferring to either the top or bottom glass does not matter).

The image of four different shapes of microwell arrays transferred onto the glass substrate using the stamping method is shown in Figure 3. One pair of cells is expected to be trapped in each of the microwells because the diameter of each cell is approximately 20 µm (the size of the well is 1250 µm^2^, 625 µm^2^ for each cell, so it is hard to trap more than two cells in each well). To transfer the microwell arrays from the PDMS stamp to the glass substrate, homemade tearing equipment was used to exert a force onto the top glass substrate. It is important to keep the peeling direction the same and while the force was continually applied, the stamp was peeled away as shown in Figure 4. As a result, the microwell arrays on the PDMS stamp were fractured and torn along a mechanically weak corner and stable PDMS patterns were transferred from the PDMS stamp to the bottom glass substrate. Naturally, the PDMS microwell arrays were transferred onto the top glass surface due to the fact that the bonding force between the PDMS and the bottom glass is larger than that which is between the PDMS microstructure and the top glass.

**Figure 5:**
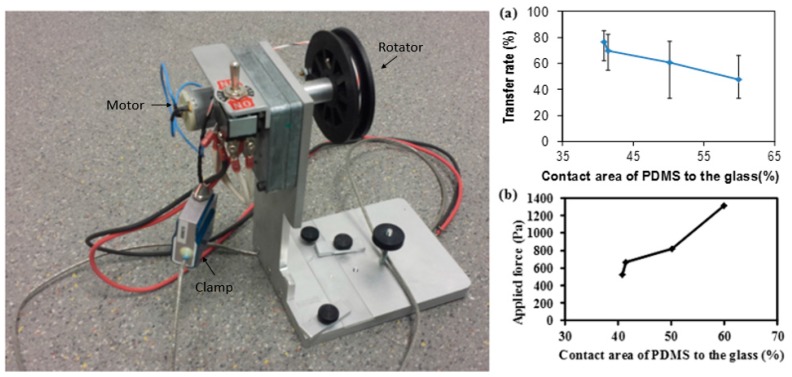
Homemade equipment for lifting PDMS (on the **left** side) and its power range (with relation of force to PDMS transfer success rate). Error bars are the range between the maximum value and minimum value; the points indicate average values. The force is applied through the clamp which is connected to a point on the glass substrate, as shown in Figure 4. (**a**) The relationship between transfer success rates in terms of contact area of the PDMS to the glass (in percentage). This shows a higher success rate when the contact area is smaller, but the success rate is decreased as the contact area increases. As the contact area increases, the bonding force between the PDMS and the glass becomes stronger and leaves a large residue. (**b**) The relationship between the applied forces to lift the PDMS to the contact area of the PDMS to the glass. A higher contact area requires more applied force to lift the PDMS since the bonding strength is stronger at a higher contact area.

As shown in Figure 5a, the transfer rate was measured as a function of the contact area of the PDMS stamp to the glass substrate using the stamp with a size of 5 mm × 5 mm. Error bars, which are the range between the maximum value and minimum value, were obtained through repeating the same experiment 10 times; the points indicate average values. The transfer rate is defined as the transferred number of patterns divided by the initial number of PDMS patterns. Since PDMS is a flexible material, the variation in the transfer rate is large. However, the average was above 50%, in the range of 40.82% to 59.91%, and the maximum was 76% at the low contact area of the PDMS to the glass. It shows that the patterns with a smaller contact area are more easily transferred since the crack resistance is low; thus, the crack starts more uniformly along the edge of the patterns at a mechanically unstable point. However, with increasing the contact area, the success rate was gradually decreased because a higher contact area leads to a stronger bonding force; this causes the PDMS stamp to be broken. As a result, more PDMS residue is left on the glass.

Moreover, the force required to lift the PDMS was measured as a function of the contact area and the result is shown in Figure 5b. As the contact area is higher, a greater area is bonded to the glass, and therefore a stronger force is required to lift it from the PDMS which can break the PDMS stamp itself.

**Figure 6:**
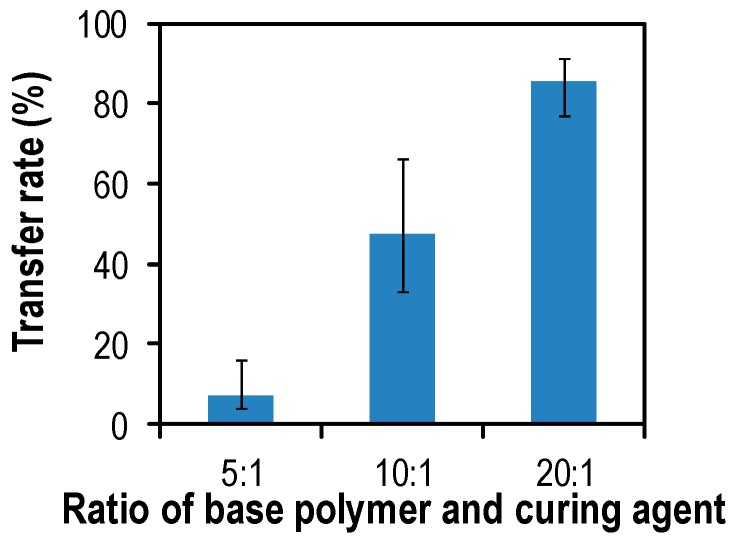
Rate of microwell arrays transferred to the glass as a function of the ratio between the pre-polymer and the curing agent. A lower amount of the curing agent in the PDMS mixture shows a better transfer rate. Error bars are the range between the maximum value and minimum value; the points indicate average values.

The stiffness of the PDMS stamp can be controlled by adjusting the mixing ratio between the pre-polymer and the curing agent. Higher ratios of the curing agent lead to the increased stiffness and hardness of the PDMS, mainly caused by an increase in cross-linking [13]. The mixing ratios (pre-polymer:curing agent) varied from 5:1 to 20:1 and the transfer rate was measured as described previously. Figure 6 shows the transfer rate as a function of the ratio between the pre-polymer and the curing agent. The error bars were obtained through repeating the same experiment 10 times. The contact area of the PDMS to the glass was fixed at 60%. Young’s modulus is defined by Hooke’s law *E* = σ/ε, where σ is an applied stress and ε is a resultant strain [14]. For the same strain, smaller strains indicate a higher stiffness of the material. At the low cross-linking density (20:1), the transfer rate was increased to 85% since the PDMS can be easily elongated, causing the crack at the corner of the patterns. Also, a smaller amount of curing agent in the PDMS has a better transfer rate must be caused by cohesion of the different polymer mixture. A smaller amount of curing agent must lead to lower cohesion of the polymer, which allows for easier breaking.

**Figure 7:**
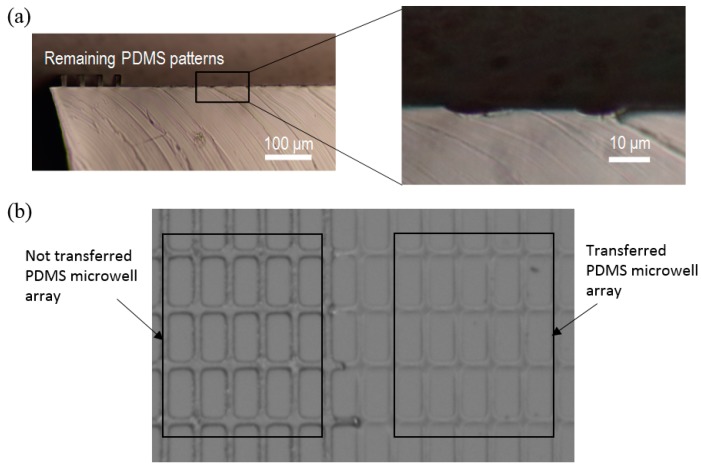
Image of microwell array on the PDMS stamp, with the transferred part and not transferred part. (**a**) Cross-section view of the microwell array on the PDMS stamp. After the transfer, the PDMS stamp is cut to calculate the uniformity of the height. (**b**) Top view of the microwell array. The left part is the microwell array that is not transferred to the PDMS microwell array, which means that the array remained on the PDMS stamp and the right part is the transferred microwell array, which means the array was removed from the stamp by being peeled off.

Next, we characterized the uniformity in the height of the microwells. As shown in Figure 7, the PDMS patterns from the stamp after the transfer were cut, and the uniformity of the pattern height was observed. Uniformity of the transferred patterns from the PDMS stamp was measured by the following definition: *U* = (*H*_max_ − *H*_min_)/*H*_mean_ × 100, where *H*_max_ is the maximum height, *H*_min_ is the minimum height and *H*_mean_ is the average value of the height. A smaller *U* value means more uniform patterns. The calculated uniformity of the patterns by this definition was 15.3%. Because of the flexible nature of PDMS, the height of the transferred PDMS microwells is not uniform but this is still enough for trapping cells in a cell-to-cell study since the height of the cell is 20 μm and the well height is close to this (usually higher than 20 μm).

**Figure 8:**
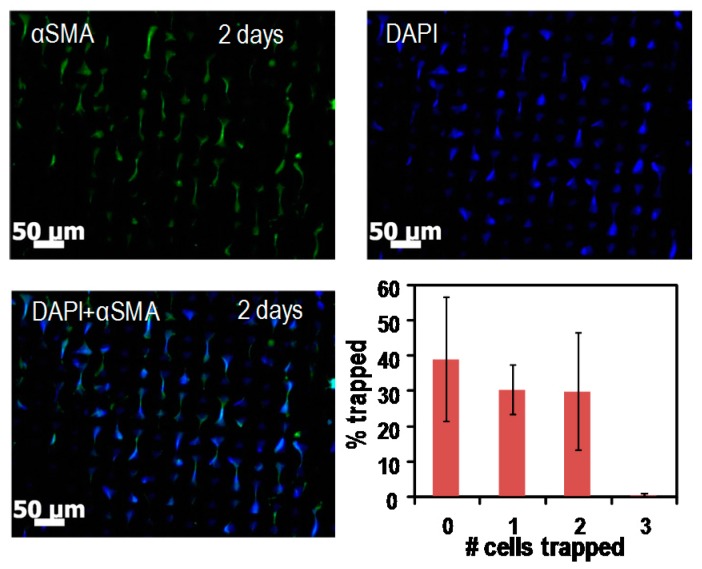
Image of the trapped cells on the bowtie-shaped microwell arrays. Cells are loaded into microwells so that one pair of cells is cultured within the microwell arrays, resulting in contacting a single neighboring cell. The cell-to-cell adhesion can be examined by immunostaining various cadherin molecules which control BM-MSCs’ (bone marrow mesenchymal stem cells) differentiation towards smooth muscle lineage. The number of cells trapped within the bowtie-shaped microwell arrays was measured.

We observed the trapped cells on the bowtie microwell arrays as shown in Figure 8. The capture efficiency for cell pairs was 29% on average. The cell-to-cell adhesion could be examined by staining cells using fluorescent dye, targeting various cadherin molecules that control BM-MSCs differentiation towards smooth muscle lineage. Specifically, we studied levels of Cad-11 (cadherin-11) and its effect on smooth muscle genes αSMA (α-smooth muscle actin), CNN-1 (calponin) and MYH11 (myosin heavy chain). Moreover, this tool is expected to be used in further studies of three or four neighboring cells in microwells; therefore, it may help to investigate biological questions such as whether cadherin-11 is a master regulator of BM-MSCs for smooth muscle differentiation.

Another possible application of the method is the fabrication of glass-PDMS-glass microfluidic devices. Since glass is a good substrate for studying cells, it is thus advisable to have a microfluidic device made by glass only, but it requires extensive work and expensive equipment. Therefore, instead of using only glass, a device made by top and bottom glass slides sandwiching a PDMS microfluidic channel layer (glass-PDMS-glass configuration) is easier and cheaper, and also is a good environment for microfluidic cell studies. For the fabrication of the glass-PDMS-glass configuration device, instead of transferring microwell arrays, we transfer the microfluidic channel pattern on a glass substrate and seal the channel with another glass substrate on top of the transferred microfluidic channel pattern. Proving the reliability of the proposed method in the glass-PDMS-glass configuration for studying cells in microfluidic systems requires good experimental results [15].

## 4. Conclusions

We have demonstrated the simple fabrication method of the microwell arrays on the glass substrate using the conventional photolithography technique. For cell-to-cell adhesion to smooth muscle cells, four different types of patterns can be successfully created on the glass substrate by tearing the patterns from the PDMS stamp to the glass substrate. The pattern transfer rate was investigated as the function of the contact area of the PDMS stamp to the glass substrate and the ratios between the pre-polymer and the curing agent. The maximum transfer rate of 76% was achieved at the ratio of 10:1 as the contact area decreased. At the ratio of 20:1, the transfer rate increased to 85%.

## Figures and Tables

**Table 1:** PDMS (polydimethylsiloxane) microwell arrays for cell-to-cell adhesion.

Shape	Rectangle	Bowtie	Wide Rhombus	Rhombus
Density of pattern (#/mm^2^)	937.11	797.20	641.46	946.84
Contact area of PDMS (polydimethylsiloxane) (%)	41.43	50.18	59.91	40.82
